# Environmental management education using immersive virtual reality in asthmatic children in Korea: a randomized controlled study (secondary publication)

**DOI:** 10.3352/jeehp.2022.19.15

**Published:** 2022-07-11

**Authors:** Seung Hyun Kim, Sang Hyun Park, Insoon Kang, Yuyoung Song, Jaehoon Lim, Wonsuck Yoon, Young Yoo

**Affiliations:** 1 Allergy Immunology Center, Korea University, Seoul, Korea; 2 Department of Pediatrics, Korea University College of Medicine, Seoul, Korea; 3 Environmental Health Center, Korea University Anam Hospital, Seoul, Korea; Hallym University, Korea

**Keywords:** Asthma, Child, Personal satisfaction, Self-management, Virtual reality

## Abstract

Awareness of environmental control is considered a significant influence on the performance of asthma self-management behaviors, which are involved in maintaining effective asthma control. This study aimed to investigate whether immersive virtual reality (VR) education is effective in environmental control education for asthmatic children in Korea. Thirty asthmatic children aged 9 to 13 years with aeroallergen sensitization were enrolled. Environmental control education for asthmatic participants was performed using immersive VR (VR group) or conventional leaflets provided by asthma specialists (control group). Five questionnaires, on awareness of environmental control, memory, assessment of intent to act, a satisfaction test, and an Asthma Control Test (ACT), were used to estimate the effects of education. The scores for awareness of environmental control, memory, and intent to act significantly increased after education in both groups, and the scores remained high until 4 weeks after education. Both groups’ ACT scores were consistently high before and 4 weeks after education. Satisfaction scores were very high in the VR group. The increased scores in awareness of environmental control and intent to act indicate that the environmental control education using VR is worthy of attention as an effective educational tool for asthma management. Further developed techniques, including active environmental interventions by participants in VR, could be applied to effective asthma management.

## Introduction

### Background/rationale

Environmental management education in asthma patients usually aims to raise awareness of the disease, manage the environmental factors that cause it, and cultivate good lifestyle habits. Until now, it has been mainly composed of verbal education by medical staff in the clinic, the provision of printed materials such as booklets, or collective presentation-type education using educational slides. However, education through oral explanations or booklets for pediatric asthma patients induces less compliance than in adults in terms of concentration and comprehension, so patients experience a lack of intent to act for proper environmental management or overcome diseases. Furthermore, a high asthma-related knowledge score does not necessarily mean good asthma management adherence or outcomes [1]. In other words, it is necessary to provide education that can be experienced in a scenario similar to real life and can be put into action immediately, rather than merely transferring knowledge.

Recently, interest in virtual reality (VR) and its use has increased. VR technology simulates space and objects built virtually through computer hardware and software to experience situations that cannot be directly experienced in the real world through the human senses of sight, hearing, touch, and smell [2]. It has been shown that there is a learning effect of VR through experiential education as well as academic achievement [3,4]. More published case studies of VR education have shown educational effects that lead to correct practical actions rather than simple knowledge transfer [5]. However, there are very few studies on the effects of learning content that can be interesting and remembered for a long time for pediatric disease prevention and treatment. In Korea, only a few studies on VR education have investigated the effect on cognitive function, balance, and memory in the elderly with mild cognitive impairment [6,7]

### Objectives

This study aimed to investigate the effectiveness of education through immersive VR experience in managing environmental factors essential for preventing and controlling children’s asthma. Specifically, environmental management awareness, environmental management memory, environmental management intent to act, and asthma control status were evaluated after the education. Furthermore, participants’ satisfaction with the education was evaluated after the intervention.

## Methods

### Ethics statement

This study was approved by the Institutional Review Board of Korea University Anam Hospital (2020AN0304). Informed consent was obtained from the parents of the participating children.

### Trial design

This was a randomized controlled study. It was described according to the CONSORT (Consolidated Standards of Reporting Trials) guideline (http://www.consort-statement.org).

### Eligibility criteria for participants, settings, and location

The eligible participants were 9- to 13-year-old children who were diagnosed with asthma from July 1, 2020, to April 30, 2021, through clinical symptoms of asthma, lung function tests, and allergy skin tests at a pediatric allergy clinic at the Korea University Anam Hospital. Thirty subjects who understood the purpose of this study voluntarily agreed to participate in this study. There were no exclusion criteria. All participants were sensitized to at least one inhalant allergen according to the skin prick test, so environmental management education was required.

### Interventions

#### Immersive VR content development

For this experiment, the authors developed content to convey how to explain and manage asthma-related environmental allergens through graphics and commentary. For about 15 minutes, environmental allergy factors were explained. For example, in the case of indoor allergens such as house dust mites, the educational material graphically displays places where there are many habitats, such as carpets and curtains inside the house, and how to act for management. We developed graphics and content to teach participants how to explain and manage mold, animal dander, cockroaches, outdoor pollen, and other factors. This content was revised and supplemented based on the solicited opinions of pediatric asthma experts, and then the final program was produced (Supplements 1, 2).

#### Environment management VR education and education through a booklet

**Fig. 1.**
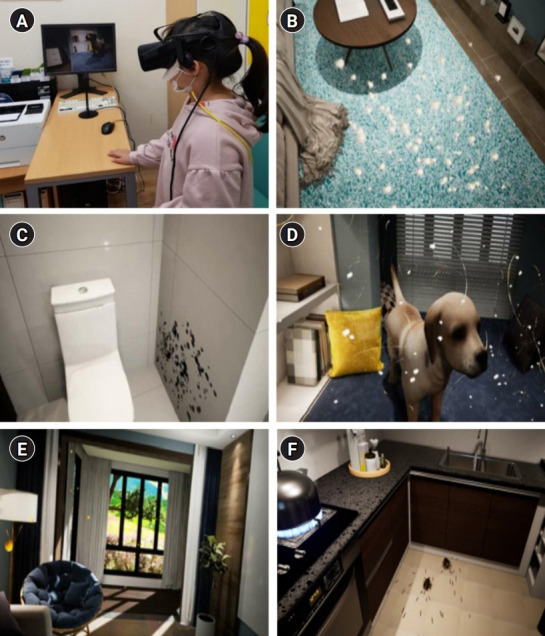
(A–F) VOCULUS RIFT DK2 Head Mount Display virtual reality system and screen.

The immersive VR education was taught by sitting in front of a computer using an OCULUS RIFT DK2 Head Mount Display (HMD) (Facebook Technologies LLC., Menlo Park, CA, USA) system. The shape of the screen that appears is shown in Fig. 1. HMD, a device used to explore VR, is a display device worn on the user’s head that completely blocks the external environment and vividly transmits images, videos, and voices to make them feel and immerse themselves in VR [8]. The control group received a verbal explanation from an asthma medical professional for the same amount of time using printed materials used for environmental management education for asthma patients in the clinic. The education time was 15 minutes for both the VR and control groups.

### Outcomes

#### Educational effectiveness evaluation survey

Questionnaires were administered immediately before, immediately after, and 4 weeks after training. The asthma environmental management awareness questionnaire was evaluated on a 3-point scale of “yes/no/don’t know” with 5 items related to asthma and the environment. The environmental management memory evaluation questionnaire consisted of 10 items. Among the 10 items, 2 items were presented as follows: “select all the substances that cause asthma” and “select all substances that aggravate asthma.” The remaining 8 items were answered on a binary scale (yes/no). The environmental management intent to act questionnaire consisted of 10 items to evaluate how strong the intent to act for environmental management was. It consisted of a 5-point Likert scale of “not at all/disagree/moderate/yes/very much.” The higher the value, the stronger the intention to act (Supplement 3). Lastly, a satisfaction survey was administered only once after education in the VR group. It consisted of a 5-point Likert scale; the higher the score, the higher the satisfaction level (Supplement 4).

#### Assessment of asthma control status

Asthma control status evaluation is an objective tool to evaluate clinical control status, and the Asthma Control Test (ACT) was used for participants aged 12–13 years [9]. For participants aged 9–11, the Childhood-ACT (C-ACT) was used. The questionnaire consisted of a total of 7 items: 4 items with scores of 0–3 points, answered by the patient, and the other 3 items with scores of 0–5 points, answered by the patient’s parents. Asthma control status was evaluated twice: before and 4 weeks after the education. This measurement tool was allowed to be used by GSK Korea (https://kr.gsk.com/ko-kr).

### Sample size

The sample size was not estimated. Only participants whose parents agreed to participate in this study were included.

### Randomization

Participants were randomly assigned to the VR group (n=15) and the control group (n=15) in equal numbers. The participants were allocated in each group sequentially after a number was marked. Participants with an odd number were allocated to the VR group, while those with an even were allocated to the control group.

### Blinding

Participants were not blinded.

### Statistical methods

The study participants’ general characteristics and questionnaire scores were expressed as mean±standard deviation (SD) or frequency. The independent sample t-test was used to analyze differences between means in 2 groups, and repeated-measures analysis of variance was used to analyze differences between means before, immediately after, and 4 weeks after training. IBM SPSS ver. 22.0 (IBM Corp., Armonk, NY, USA) was used for analysis.

## Results

### Participants

Of the 30 participants, 17 (56.7%) were boys, and the mean±SD age was 12.5±2.6 years. The average duration of asthma prevalence was 3.2±2.7 years, and among their parents, 4 (26.7%) were diagnosed with asthma. There were no significant differences in the blood total immunoglobulin E concentration, peripheral blood eosinophil fraction, eosinophil cation protein concentration, and baseline lung function between the VR group and the control group. There were also no significant differences in the exhaled nitric oxide concentration and methacholine airway hypersensitivity between the 2 groups (Table 1).

### Outcomes and estimation

#### Allergen sensitization profile

Table 2 shows the allergen sensitization profile of the participants’ skin prick tests. All 30 participants were positive for 1 or more inhalant allergen groups. By allergen group, positive results were obtained for house dust mites (25 [83.3%]), mold (5 [16.7%]), animal dander (15 [50.0%]), pollen (15 [50.0%]), and cockroaches (2 [6.67%]), and there was no significant difference in the types of allergen sensitized between the 2 groups.

#### Effect of environmental management education

**Fig. 2.**
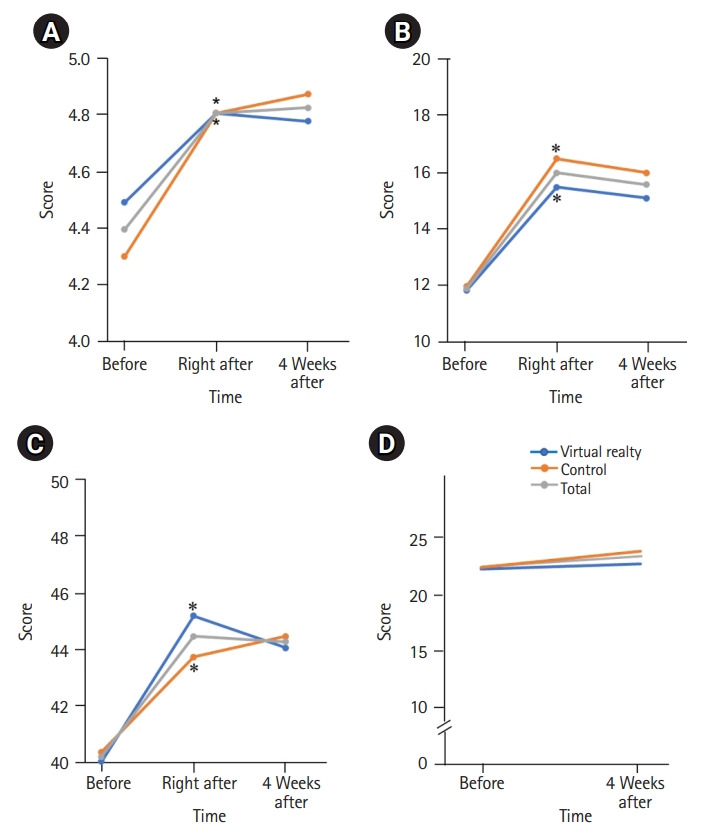
Awareness of environmental control (A), memory (B), intent to act scores (C), and Asthma Control Test results (D). *P<0.05 compared to the before education result.

As shown in Fig. 2, both groups significantly improved in environmental management awareness, memory, and intent to act immediately after training; these scores remained high even 4 weeks after the training. However, there was no significant difference in the scores of the environmental management awareness (Fig. 2A), memory (Fig. 2B), and intent to act for environmental management (Fig. 2C) conducted before, immediately after, and 4 weeks after education between the 2 groups. Educational satisfaction was evaluated only in the VR group, and the mean±SD satisfaction score was an average of 4.40±0.28, out of 5 points for “interest, adaptability to virtual reality, knowledge of allergy disease management, help in understanding environmental management contents, and willingness to re-participate in VR education.” The raw response data file is available from Dataset 1.

#### Assessment of asthma control status

Asthma control status was measured using the ACT or C-ACT questionnaire according to the age of each subject, and there was no significant improvement in the scores before and 4 weeks after training. There was no significant difference between the 2 groups (Fig. 2D).

### Ancillary analyses

None.

### Harms

None.

## Discussion

### Interpretation

**Fig. 3.**
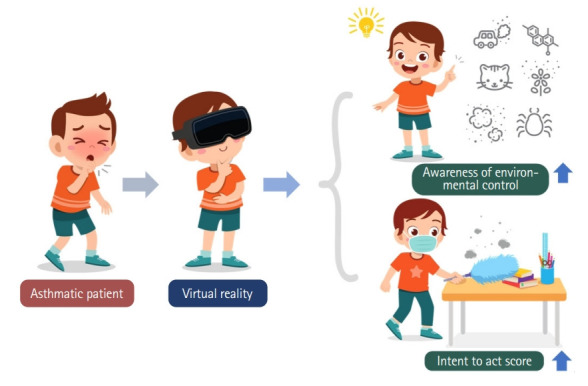
Environmental management education using a virtual reality program (graphical abstract).

In this study, we developed immersive VR content for asthma environment management education and compared the asthma environment management awareness, memory, and intent to act for environmental management. Furthermore, asthma control status between the experimental and control groups was compared. Both groups showed higher awareness, memory, and intent to act in environmental management after education than before, which continued to be high even 4 weeks after the education (Fig. 3). Satisfaction with the VR education method was very high, and it seems that the participants were interested in and understood the new VR education, which motivated them to practice.

VR is divided into non-immersive and immersive types according to the experience method. The non-immersive type involves experiencing the virtual world through an image output device such as a monitor or projector without wearing a separate device, while the immersive type refers to VR in which the user experiences wearing an HMD connected to a computer or mobile device [10]. The immersive VR used in this study seizes the user’s field of view, blocking visual stimuli from the real world, and the user only receives 3-dimensional image output from the HMD. Through this, it is possible to experience the virtual world like the real world, and a strong sense of presence and immersion is induced [11]. This study showed no difference in scores by gender and age. Anyone can use it, as the participants did not complain of any significant inconvenience.

The participants in the VR group said that they continued to manage their environment because they were interested in themselves, even though no additional education was conducted within 4 weeks after the initial training. The improvement in the intent to act score also helped foster the intention to act by supporting the participants to empathize with interest by educating them using VR on environmental management topics they had never encountered.

The environmental management program developed in this study allows participants to receive education without the help of medical specialists. This asthma environment management VR content development is still an early attempt. Better educational effects and disease prevention performance can be shown if interventions are developed with higher quality and utilized based on a more sophisticated theoretical framework to improve the disease control status and awareness of patients in the future. In this study, participants improved their awareness of long-term asthma environment management and their memory after training.

Both the VR and control groups showed mostly well-controlled scores in the asthma control status evaluation before and 4 weeks after education using C-ACT or ACT, respectively. There were no significant differences between the 2 groups, or before and after education within the 2 groups. An explanation for this is that most of the original participants with asthma were well controlled, so it is thought that the score did not increase significantly after education.

### Comparison with previous studies

Until now, only a few research results for functional game development [12] or patients with mild cognitive impairment have been reported in research on VR used for health and medical care in Korea [6,7], but active research has not yet explored environmental management education targeting patients such as those with allergic asthma. Until now, education for asthma patients has mainly been provided through picture booklets [13] or small group education [14] in hospitals, which showed positive results such as improvement in self-management ability and improvement of self-efficacy. However, these educational methods are usually small-scale or require the help of trained medical professionals, so there is a limit to continuous and large-scale use. VR learning positively affects learning outcomes, with its relatively high sense of presence and immersion compared to conventional education methods [15].

### Limitation

In this study, participants did not complain of dizziness, discomfort when wearing the headset, or difficulty in using the equipment during the education period, but participants who were new to VR education spent a lot of time looking around the background rather than focusing on the important educational content. Therefore, the background may have been a bit distracting, which is something to be improved. Other limitations include the fact that the development of VR content requires the help of a technical development expert and takes time; it is also necessary to develop objective indicators to evaluate the educational effect and completely block external stimuli.

### Generalizability

Since the results of this study were obtained from pediatric patients at a single institution, it is unknown whether they can be applied to patients from other institutions overseas or in Korea. It is necessary to verify this method through a multinational, multicenter study.

## Conclusion

There were no significant differences in the scores for environmental management awareness, memory, and intent to act for environmental management conducted before, immediately after, and 4 weeks after education between the participants in VR education wearing HMDs and those using printed materials. The satisfaction score for VR education was high. In other words, asthma patients quickly acquired environmental management knowledge, remembered it for a long time, and showed a strong will to practice. The results of this study can be used as primary data to strengthen the theoretical foundation of VR education, improve the learning effect through quantitative and qualitative education, and positively affect disease management.

## Figures and Tables

**Table 1.** Clinical characteristics of study participants

Characteristic	Virtual reality (n=15)	Control (n=15)	P-value
Age (yr)	11.7±2.5	13.2±2.6	0.123
Sex			0.070
Boy	6	11	
Girl	9	9	
Immunoglobulin E (IU/mL)	188.7 (38.5–925.2)	235.1 (68.7–804.3)	0.686
Eosinophils (%)	2.29 (0.64–8.25)	3.10 (1.77–5.42)	0.402
Eosinophil cationic protein (μg/mL)	14.4 (4.66–44.7)	8.58 (3.16–23.3)	0.196
FEV1 (%pred)	92.1±9.3	91.4±12.6	0.290
FVC (%pred)	90.7±10.7	96.7±17.6	0.255
FEV1/FVC (%)	88.2±8.3	85.3±7.3	0.313
FEF25–75 (%pred)	100.4±33.2	91.9±23.1	0.424
FeNO (ppb)	15.0 (6.23–36.2)	15.2 (7.39–31.2)	0.955
MChPC20 (mg/mL)	3.94 (0.76–20.3)	5.10 (0.76–34.5)	0.694

Values are presented as mean±SD, number, or geometric mean (range of 1 SD). FEV1, forced expiratory volume in 1 second; FVC, forced vital capacity; FEF25–75, forced expiratory flow between 25% and 75% of FVC; FeNO, fractional exhaled nitric oxide; MChPC20, provocative concentration of methacholine causing a 20% decline in FEV1; SD, standard deviation.

**Table 2.** Prevalence of positive skin prick test results in the study participants

Variable	Virtual reality (%)	Control (%)	P-value
House dust mites	13 (86.7)	12 (80.0)	1.000
Mold	1 (6.7)	4 (26.7)	0.330
Animal dander	7 (46.7)	8 (53.3)	1.000
Pollen	6 (40.0)	9 (60.0)	0.233
Cockroaches	0	2 (13.3)	0.241

## Footnotes

**Data availability**
Data files are available from Harvard Dataverse: https://doi.org/10.7910/DVN/8JN3F7 Dataset 1. Participants’ response data to the survey on the awareness of environmental control, the ability to remember educational content, the assessment of intent to act, and their satisfaction with virtual reality.

## Supplementary materials

Data files are available from Harvard Dataverse: https://doi.org/10.7910/DVN/8JN3F7

Supplement 1. Video of education on allergens for an asthma patient who wore a head mount display.

Supplement 2. Screen view of an asthma patient who wore a head mount display during the education on allergens.

Supplement 3. Survey items on awareness of environmental control, ability to remember educational content, and assessment of intent to act in study participants (in Korean).

Supplement 4. Satisfaction survey items in virtual reality (in Korean).

Supplement 5. Audio recording of the abstract.
